# Bullatine A exerts anti-inflammatory effects by inhibiting the ROS/JNK/NF-κB pathway and attenuating systemic inflammatory responses in mice

**DOI:** 10.1080/13880209.2022.2121410

**Published:** 2022-10-06

**Authors:** Shuhan Liu, Na Che, Wen Ou, Meichen Yan, Yajin Liao, Yong Cheng

**Affiliations:** a College of Life and Environmental Sciences, Center on Translational Neuroscience, Minzu University of China, Beijing, China; b Key Laboratory of Modern Preparation of TCM, Ministry of Education, Jiangxi University of Traditional Chinese Medicine, Nanchang, China; c Department of Neurology, The Second Affiliated Hospital, Hengyang Medical School, University of South China, Hengyang, PR China; d The Brain Science Center, Beijing Institute of Basic Medical Sciences, Beijing, China; e Institute of National Security, Minzu University of China, Beijing, China

**Keywords:** *Aconiti brachypodi* Radix, inflammation, LPS, BV-2, macrophage, MAPKs

## Abstract

**Context:**

*Aconiti brachypodi* Radix (Xue-shang-yi-zhi-hao) is a traditional Chinese herbal medicine that is capable of anti-analgesic and anti-inflammatory effects. Bullatine A (BA) is one of the major active ingredients of this plant, and most of the previous studies reported that it has anti-analgesic effects. However, the mechanism of BA anti-inflammatory remains unclear.

**Objective:**

This study investigates the anti-inflammatory activities of BA, both *in vitro* and *in vivo*, and elucidates its mechanism.

**Materials and methods:**

*In vitro*, BA (10, 20, 40 and 80 μM) was added to 1 µg/mL of lipopolysaccharide (LPS)-activated microglia BV2 cells and immortalized murine bone marrow-derived macrophages, respectively. After 6 h, the mRNA and protein levels of inflammatory factors were determined by real-time quantitative PCR and western blotting. *In vivo*, C57BL/6 mice were randomly divided into control, model (5 mg/kg dose of LPS) and treated groups (LPS with 5, 10 or 20 mg/kg dose of BA) to evaluate the anti-inflammatory efficacy of BA.

**Results:**

BA significantly inhibited LPS-induced expression of inflammatory factors, such as IL-1β, IL-6, TNF-α, inducible nitric oxide synthase (iNOS) and COX-2. Further investigations showed that BA reduced the translocation of NF-κB p65 (38.5%, *p* < 0.01). BA also reduced the phosphorylation of c-Jun N-terminal kinase (JNK) (11.2%, *p* < 0.05) and reactive oxygen species (ROS) generation (24.2%, *p* < 0.01). Furthermore, BA treatment attenuated the LPS-primed inflammatory response and liver and lung damage *in vivo*.

**Conclusions:**

BA can inhibit the inflammatory response in part through the ROS/JNK/NF-κB signalling pathway, providing a theoretical basis for the clinical application of BA in the treatment of periphery inflammatory diseases.

## Introduction

Phytopharmaceuticals have gained increasing attention worldwide because of their beneficial role in treating a variety of human diseases and the presence of a large number of natural compounds with various chemical properties (Khan et al. 2018). Preparations of the *Aconitum* genus (Ranunculaceae) have been extensively used to treat and prevent multiple diseases, including pain and neurological and cardiovascular diseases in China and other Asian countries (Li J et al. 2013). In China, approximately 76 *Aconitum* species are used as medicinal plants based on their toxicity and side effects, therapeutic effects, and phytochemical properties (Ren et al. 2012; Wang and Li 2020). *Aconiti brachypodi* Radix (Xue-shang-yi-zhi-hao), the dried roots of *Aconitum brachypodum* Diels and several other morphologically similar *Aconitum* species, was listed in the Chinese Pharmacopoeia in 1977 for the treatment of rheumatism and pain (China Pharmacopoeia Committee 1977; Huang et al. 2016).

**Figure 1.**
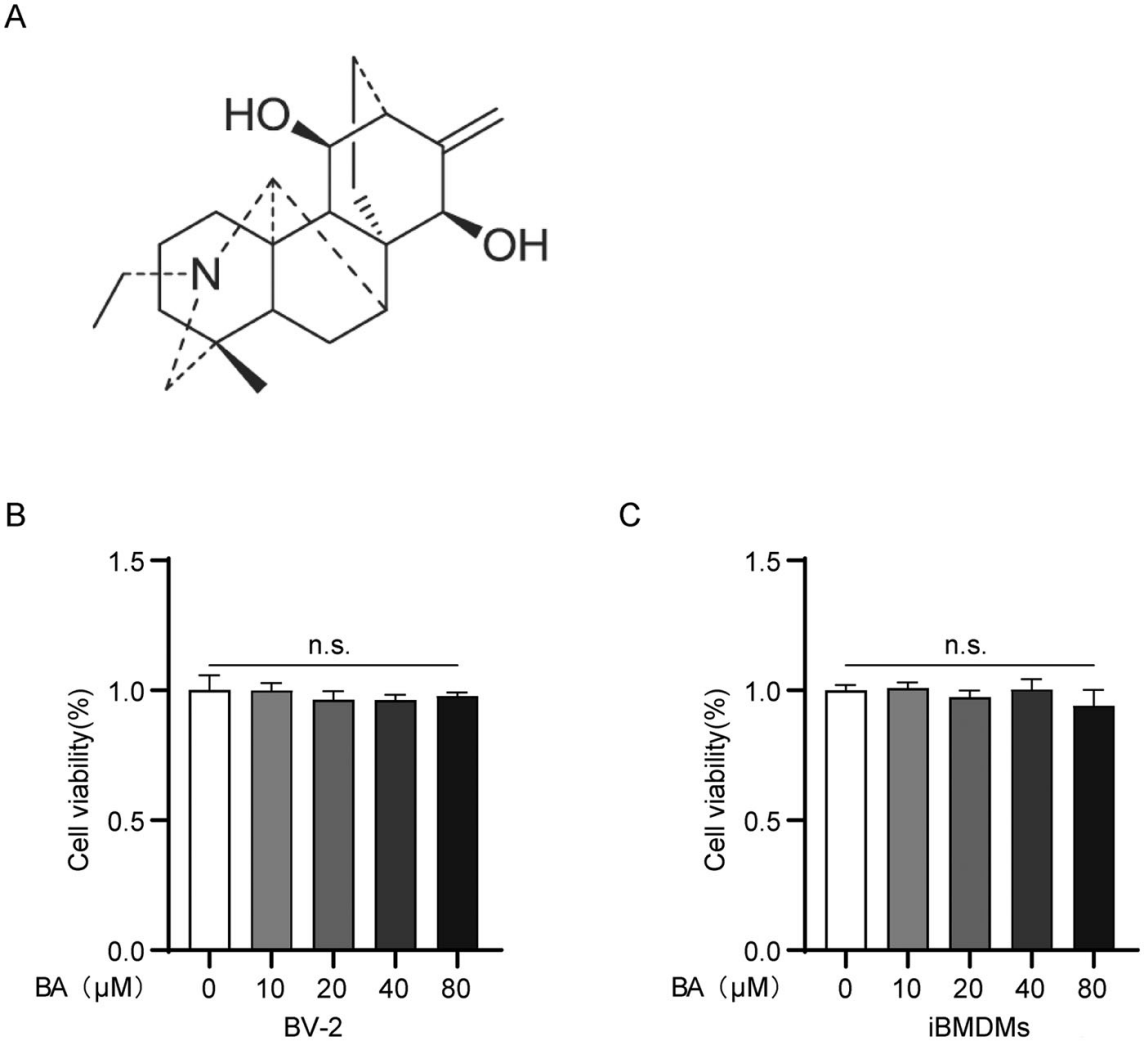
The structure of Bullatine A and the effect of BA on cell viability. (A) The chemical structure of BA. The cell viability of BV-2 (B) and iBMDM (C) cells incubated with BA in different concentrations. n.s.: no significant.

Bullatine A (BA, C_22_H_33_NO_2_, chemical formula shown in Figure 1(A)) is one of the major bioactive compounds isolated from *Aconiti brachypodi* Radix. The ethanol extract of *Aconiti brachypodi* Radix, which primarily consists of BA, has been reported to attenuate heat-, acetic acid- and formalin-induced pain responses (Ren et al. 2012). Further studies have revealed that BA is an active alkaloid compound that attenuates pain hypersensitivity in various rat pain models (Huang et al. 2016). In addition, BA inhibits ATP-induced BV-2 microglial cell death by blocking ATP-primed upregulation of the P2X receptor (Li et al. 2013), suggesting that BA may have beneficial therapeutic effects in the treatment of conditions associated with ATP-involved inflammatory responses. Furthermore, BA has the lowest cytotoxicity among the alkaloids isolated from *Aconitum* (Singhuber et al. 2009; Huang et al. 2016).

Here, we revealed that BA could inhibit lipopolysaccharide (LPS)-stimulated inflammation *in vitro* and *in vivo* and attenuate LPS-induced liver and lung injury. In addition, we found that BA treatment reduced the production of reactive oxygen species (ROS), inducible nitric oxide synthase (iNOS) protein levels and c-Jun N-terminal kinase (JNK) phosphorylation. This study suggests that BA could be used for anti-inflammatory disease therapy.

## Materials and methods

### Reagents and antibodies

BA (purity ≥98%) was purchased from Psaitong (Beijing, China). Dimethyl sulphoxide (DMSO) and LPS were purchased from Sigma-Aldrich (St. Louis, MO). Antibodies used for western blotting were as follows: anti-iNOS/NOS Type II (#610332; BD Biosciences, San Jose, CA); anti-cyclooxygenase (COX)-2 (#160106), anti-phospho-IKKα/β (S176/180, 16A6, #2697P), anti-phospho-IκBα (Ser32, #2859), anti-IκBα (44D4, #4814), anti-phospho-ERK1/2 (Thr202/Tyr204, #9101), anti-ERK1/2 (#9102), anti-phospho-p38 MAPK (Thr180/Tyr182, #4511), anti-p38 MAPK (#9212), anti-phospho-SAPK/JNK (Thr183/Tyr185, #9251), anti-JNK2 (56G8, #9258) and anti-NF-κB p65 (D14E12, #8242) (Cell Signaling Technology, Beverly, MA); anti-IKKα (CHUK, #A2062; ABclonal Technology, Woburn, MA); anti-β-tubulin (#CW0098A) and anti-GAPDH (#CW0266A) (CWBiotech, Beijing, China); and anti-histone H2B (Santa Cruz Biotechnology, Inc., Dallas, TX).

### Mice

Male C57BL/6 mice (8–10 weeks old, weight 20–25 g) were purchased from Beijing Vital River Laboratory Animal Technology Co., Ltd. (Beijing, China). Mice were housed four per cage under a 12 h light/dark cycle at 22–24 °C with free access to water and food. All experimental animal procedures were approved by the Institutional Animal Care and Use Committee of the Beijing Institute of Basic Medical Sciences (animal ethics number: syxk 2019-0004).

### Cell culture

BV-2 and immortalized murine bone marrow-derived macrophage (iBMDM) cell lines were obtained from the American Type Culture Collection (Manassas, VA) and maintained in Dulbecco’s modified Eagle’s medium (#11965-092; Life Technologies, Waltham, MA) supplemented with 10% heat-inactivated foetal bovine serum (#04-001-1A; Biological Industries, Beit Haemek, Israel) and 1% penicillin–streptomycin solution (#03-031-1B; Biological Industries, Beit Haemek, Israel) at 37 °C in a humidified atmosphere with 5% CO_2_.

### Cell viability assay

A Cell Counting Kit-8 (CCK-8) assay (ab228554; Abcam, Cambridge, UK) was used to evaluate cell viability following the manufacturer’s instructions. Briefly, cells were plated at a density of 1 × 10^5^ cells/mL in 96-well plates and then exposed to different concentrations of BA for 24 h. Subsequently, 10 μL of CCK-8 reagent was added to each well and incubated at 37 °C for an additional 2 h. Finally, a Spectra Max i3x (Molecular Devices, Sunnyvale, CA) was used to measure the optical density at 450 nm.

### Dual-luciferase reporter system

A nuclear factor-κB (NF-κB) reporter was generated in our laboratory. Briefly, the NF-κB promoter was cloned into a pGL3-luciferase reporter vector (Promega, Madison, WI). HEK293T cells were then co-transfected with a pCMV-Renilla plasmid and the NF-κB reporter using Lipofectamine 2000 transfection reagent (#11668019; Invitrogen, Waltham, MA). Twenty-four hours after transfection, the cells were lysed, and luciferase activity was measured using a dual-luciferase reporter detection system (Promega, Madison, WI).

### Measurement of intracellular ROS

Intracellular ROS were monitored by staining the cells with 2′,7′-dichlorofluorescein diacetate (DCFH-DA; Beyotime, Shanghai, China). Briefly, iBMDM cells were treated with 1 µg/mL LPS or in combination with 80 μM of BA for 24 h, then washed twice with serum-free medium to remove excess nanoparticles. The cells were then incubated with 10 μM DCFH-DA at 37 °C for 20 min and analysed using a FACS Vantage flow cytometer (BD Biosciences, San Jose, CA).

### Immunofluorescence assay

Briefly, iBMDM cells (5 × 10^5^ cells/well) were seeded on coverslips, treated with 80 μM of BA for 1 h, and then stimulated with LPS for the next 2 h. After incubation with 4% paraformaldehyde for 20 min, the cells were permeabilized with 0.5% Triton X-100 and blocked with 1% bovine serum albumin. For staining of NF-κB p65, the cells were further incubated with anti-NF-κB p65 (1:400) antibodies at 4 °C overnight, followed by incubation with a secondary TRITC-conjugated antibody at room temperature for 1 h. Nuclei were counterstained with Hoechst (1 ng/mL) for 5 min. Images were obtained using a confocal microscope (Leica Camera AG, Wetzlar, Germany).

### *In vivo* LPS challenge

C57BL/6 mice were injected intraperitoneally (i.p.) with different dosages of BA (5, 10 or 20 mg/kg) or vehicle (DMSO/saline) two times at 12 h and 1 h prior to i.p. injection of LPS (5 mg/kg) or saline, respectively. Then, the mice were sacrificed 4 h post LPS injection, and liver and lung tissues were either collected and stored at −80 °C or fixed with 4% paraformaldehyde for further study.

### Histological assessment

Liver and lung tissues were fixed in 4% paraformaldehyde and embedded in paraffin. The sections of various organs were then stained with haematoxylin and eosin (H&E). The pathological scores for the liver and lung were determined according to previous studies, with minor modifications (Maehara et al. 2020). The score was mainly determined as the degree of immune cell infiltration and structure disruption as follows: 0, none; 1, mild; 2, moderate; and 3, severe.

### Enzyme-linked immunosorbent assay (ELISA)

Blood collected from mice was coagulated at room temperature and then centrifuged for 15 min at 1500×*g* to separate the serum. The serum samples were transferred into another tube and stored at −80 °C before analysis. Serum levels of IL-6 were measured using ELISA with commercially available kits (#431304, BioLegend, San Diego, CA) according to the manufacturer’s instructions.

### Quantitative real-time PCR

Total RNA was extracted from tissues and cultured cells using TRIzol Reagent (#15596026; Life Technologies, Waltham, MA) according to the manufacturer’s instructions. Finely chopped tissues were placed into TRIzol and immediately homogenized using a homogenizer. Then, mRNA from each sample was converted to cDNA using a cDNA synthesis kit (AE311-03; TransGen Biotech, Beijing, China). Finally, quantitative PCR was performed using a SYBR Green master mix (#A304-10; GenStar, Beijing, China) with an ABI studio Q3 Real Time PCR system (Life Technologies, Waltham, MA). The primer sequences used are shown in Table 1.

**Table 1.** Primer pairs used for qPCR analysis.

Target	Sequence (5′–3′)
*β-actin*	Forward: GGCTGTATTCCCCTCCATCG Reverse: CCAGTTGGTAACAATGCCATGT
*IL-1β*	Forward: GTCGCTCAGGGTCACAAGAA Reverse: CTGCTGCCTAATGTCCCCTT
*IL-6*	Forward: GCTACCAAACTGGATATAATCAGGA Reverse: CCAGGTAGCTATGGTACTCCAGAA
*iNOS*	Forward: GTTCTCAGCCCAACAATACAAGA Reverse: GTGGACGGGTCGATGTCAC
*TNF-α*	Forward: CAGGCGGTGCCTATGTCTC Reverse: CGATCACCCCGAAGTTCAGTAG

### Western blotting

Western blot analyses were conducted as previously described (Pan et al. 2021). Briefly, tissues or cells were lysed with radioimmunoprecipitation assay buffer containing a cocktail of protease and phosphatase inhibitors. The protein was isolated by sodium dodecyl sulphate–polyacrylamide gel electrophoresis and then transferred to a polyvinylidene fluoride membrane (#ISEQ00010; MilliporeSigma, Burlington, MA). The membrane was blocked with 5% non-fat milk in Tris-buffered saline and incubated overnight with primary antibodies at 4 °C. Finally, the proteins were detected using horseradish peroxidase-conjugated secondary antibodies.

### Statistical analysis

GraphPad Prism software (version 8, La Jolla, CA) was used for the statistical analyses. All data are presented as mean ± standard error unless otherwise noted. The significance of differences was assessed using an unpaired Student’s *t*-test or one-way analysis of variance. Statistical significance was set at *p*< 0.05.

## Results

### BA inhibits LPS-induced expression of pro-inflammatory cytokines in microglia and macrophages

**Figure 2.**
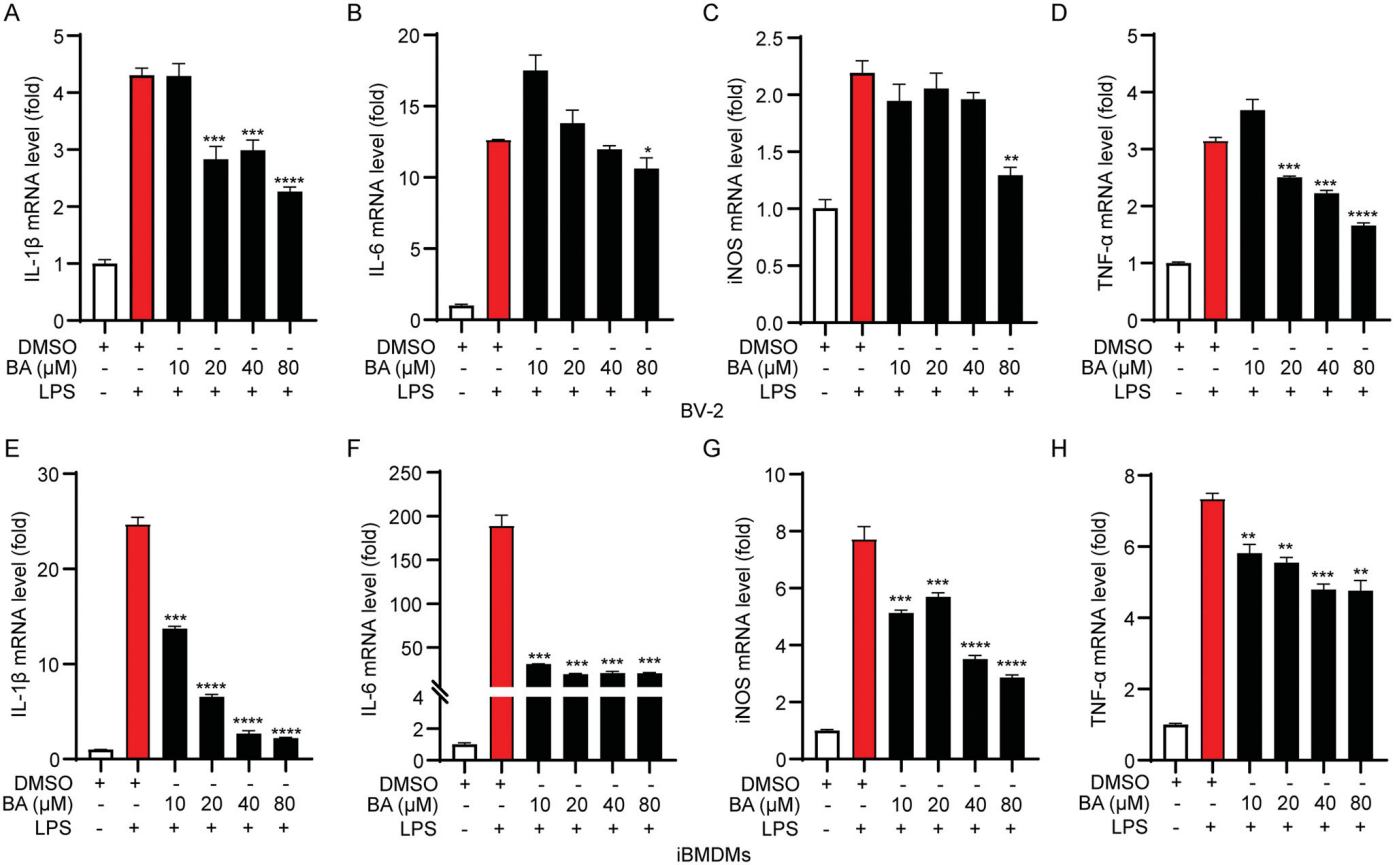
Bullatine A inhibited the expression of inflammatory genes in LPS-activated microglia and macrophages. The mRNA levels of IL-1β (A), IL-6 (B), iNOS (C) and TNF-α (D) in BV-2 cells treated with LPS (1 µg/mL) and BA (10–80 μM) for 6 h were detected by quantitative RT-PCR. The mRNA levels of IL-1β (E), IL-6 (F), iNOS (G) and TNF-α (H) in iBMDM cells treated with LPS (1 µg/mL) and BA (10–80 μM) for 6 h were detected by quantitative RT-PCR. *p< 0.05, **p< 0.01, ***p< 0.001 and ****p< 0.0001 vs. LPS treatment group.

To evaluate the potential cytotoxicity of BA, BV2/iBMDM cells were incubated with 0, 10, 20, 40 and 80 μM BA for 24 h, after which cell viability was determined. No obvious cytotoxicity was observed when cells were treated with BA at 80 μM for 24 h (Figure 1(B,C)). Thus, this concentration range was used to investigate the anti-inflammatory properties of BA *in vitro*. To further investigate the anti-inflammatory effect of BA, BV-2 cells were pre-treated with BA (20, 40 and 80 μM) for 1 h, followed by incubation with 1 μg/mL LPS for 6 h. The results showed that the mRNA levels of interleukin (IL)-1β, IL-6, iNOS and tumour necrosis factor (TNF)-α which were upregulated by LPS stimulation were all inhibited when pre-treated with BA (Figure 2(A–D)). We also examined the effect of BA on iBMDMs, immortalized macrophages that are commonly used in *in vitro* cell-based assays to examine the mechanisms of innate immune activation (De Nardo et al. 2018). Consistent with the results in the BV2 cells, BA inhibited LPS-induced transcription of IL-1β, IL-6, iNOS and TNF-α in a dose-dependent manner in iBMDMs (Figure 2(E–H)). Together, these results indicate that BA can effectively inhibit LPS-induced inflammation in both microglia and macrophages.

### BA exerts anti-inflammatory activity by blocking activation of the NF-κB signalling pathway

**Figure 3.**
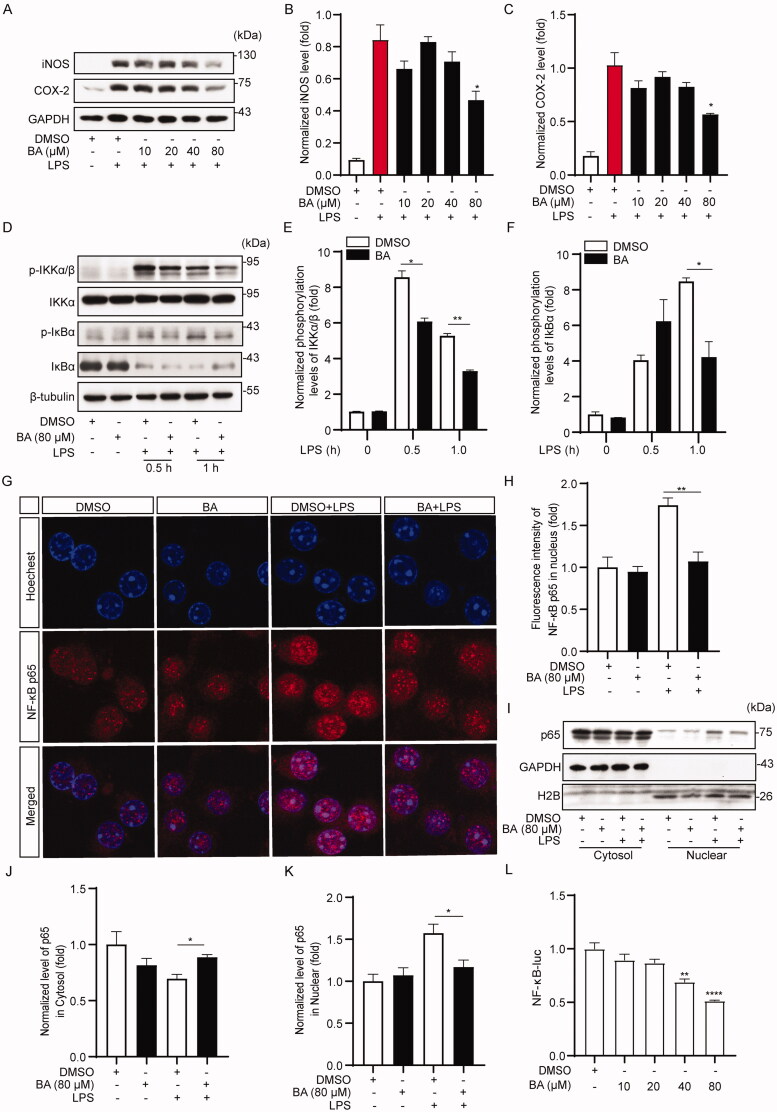
Bullatine A inhibited the activation of the NF-κB pathway. (A) Effects in BA on the high expression of iNOS and COX-2 proteins in LPS-activated iBMDM cells. (B, C) Data were standardized on the basis of GAPDH levels. (D) Effect of BA on the phosphorylation of IKKα/β and IκBα proteins detected by western blotting. (E, F) Data were standardized on the basis of percentages of levels of unphosphorylated forms (total-IKKα/β and total-IκBα). (G) Effect of BA on the translocation of NF-κB p65 into nucleus was detected by immunofluorescence assay. After BA (80 μM) treatment for 1 h, iBMDM cells treated with LPS (1 μg/mL) for 2 h. (H) Fluorescence intensity of NF-κB p65 in nucleus was analysed by Image J software. (I) Expressions of NF-κB p65 in cytosol and nuclear were measured by western blotting, GAPDH and H2B were taken as control. (J, K) Data were standardized on the basis of percentages of GAPDH and H2B levels. (L) Quantitative analysis of the effect of BA on NF-κB luciferase activity. *p< 0.05, **p< 0.01 and ****p< 0.0001 vs. LPS treatment group or DMSO treatment group.

It is well known that the NF-κB signalling pathway plays a critical role in the cellular inflammatory response, mainly manifested by the NF-κB transcription factor regulating the expression of pro-inflammatory cytokines, iNOS and cyclooxygenase-2 (COX-2) proteins, and is involved in oxidative stress and inflammation (Wang, Gu, et al. 2021). Therefore, we further determined the effects of BA on LPS-induced iNOS and COX-2 expression in iBMDM cells. The results showed that the protein levels of iNOS and COX-2 were significantly increased after LPS stimulation (Figure 3(A)) and were impaired when cells were pre-treated with BA (Figure 3(B,C)). These results further confirmed the anti-inflammatory activity of BA.

To explore the anti-inflammatory mechanisms of BA, we analysed the activation of the NF-κB pathway by western blotting. As expected, LPS stimulation markedly increased the phosphorylation levels of IKKα/β and IκBα (Figure 3(D)) that were significantly inhibited in cells pre-treated with BA, especially at 1 h post LPS stimulation (Figure 3(E,F)). In addition, the translocation of the NF-κB p65 subunit to the nucleus induced by LPS was dramatically decreased. Our immunofluorescence assay results showed that BA preconditioning greatly inhibited p65 subunit transport to the nucleus at 2 h post LPS stimulation (Figure 3(G,H)). The results were further analysed by a nucleus/cytoplasm separation assay followed by western blotting. LPS exposure resulted in the translocation of p65 from the cytosol to the nucleus, whereas a significant reduction in nuclear p65 protein and a considerable increase in cytoplasmic p65 protein was observed in cells pre-treated with BA (Figure 3(I–K)). Next, using a dual-luciferase reporter system, we found that NF-κB-promoter luciferase activity was significantly decreased when the concentration of BA reached 40 μM (Figure 3(L)). These results indicate that BA exhibits anti-inflammatory effects *in vitro*, probably by blocking the activation of the NF-κB signalling pathway.

### BA inhibits activation of the NF-κB pathway by impairing JNK phosphorylation

**Figure 4.**
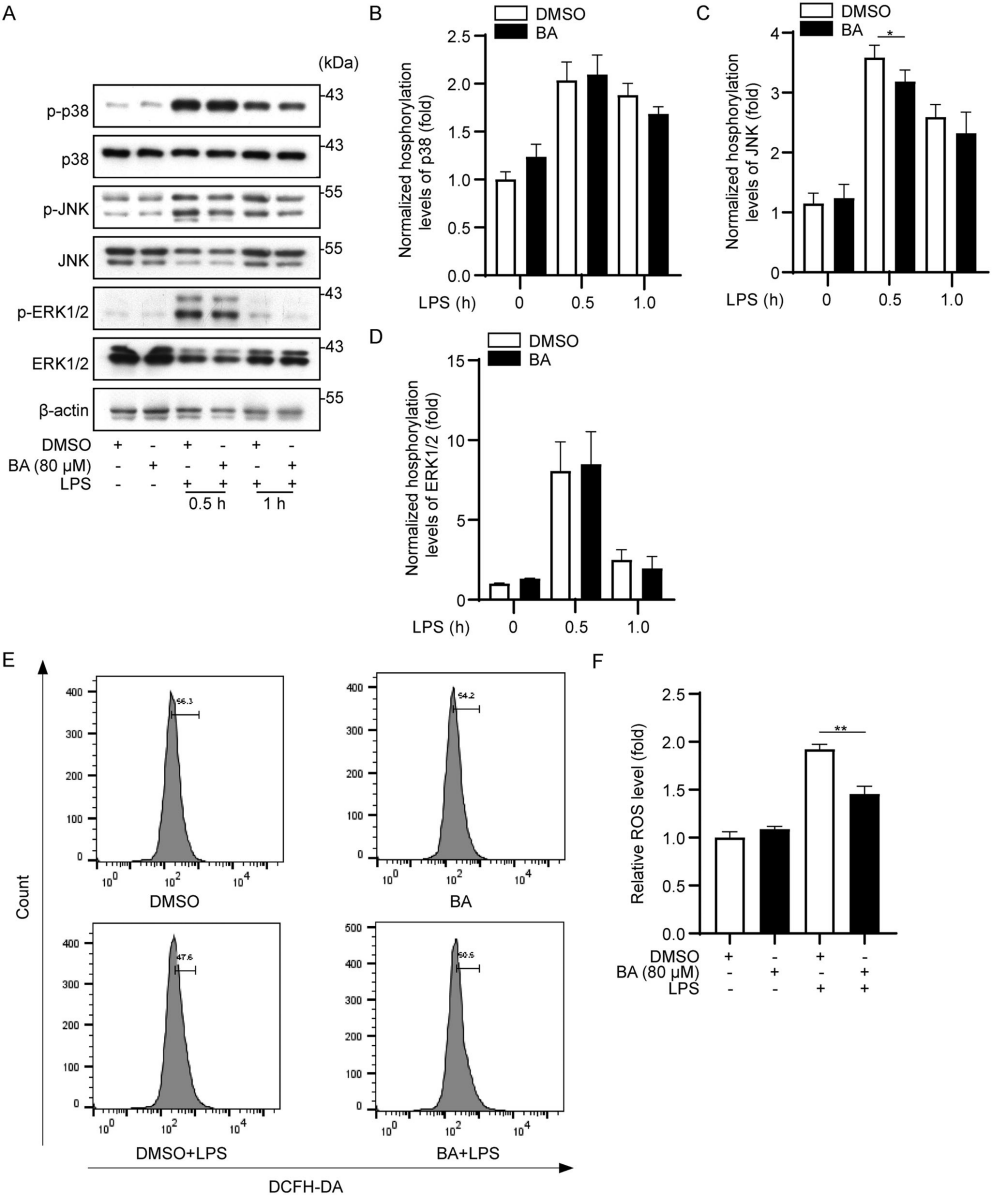
Bullatine A inhibited JNK phosphorylation and intracellular ROS production. (A) Effect of BA on the phosphorylation of p38, JNK and ERK proteins detected by western blotting. (B–D) Densitometric analysis data were standardized on the basis of percentages of levels of unphosphorylated forms (total-p38, total-JNK and total-ERK). (E) Effects of BA on the production of ROS in LPS-stimulated iBMDM cells. Cells were pre-treated with BA at 80 μM for 1 h and then treated with LPS at 1 μg/mL for 24 h. (F) Data were standardized on the basis of ROS levels in the control group. *p< 0.05 and **p< 0.01 vs. LPS treatment group.

Mitogen-activated protein kinases (MAPKs) play an important role in extracellular signal transduction in cellular responses (Wang, Gu, et al. 2021). Therefore, we explored the effect of BA on the MAPK signalling pathway. Western blot analysis showed that the phosphorylation levels of major proteins of MAPK pathways, including p38, JNK and ERK1/2, were significantly increased after 0.5 or 1.0 h stimulation by LPS in iBMDMs. Furthermore, pre-treatment with BA significantly inhibited the phosphorylation of JNK, but did not show a remarkable inhibitory effect on the phosphorylation of p38 and ERK1/2 (Figure 4(A–D)). These results indicate that BA may inhibit activation of the NF-κB pathway by impairing the phosphorylation of JNK.

### BA reduced LPS-induced stimulation of ROS

It has recently been recognized that JNK can be activated by ROS, further facilitating LPS-induced inflammation (Hensley et al. 2000; Traba and Sack 2017). Therefore, we measured intracellular ROS levels using DCFH-DA staining and flow cytometry to investigate whether BA could inhibit ROS production. The results showed that the production of ROS was significantly increased after LPS stimulation; however, pre-treatment with BA markedly attenuated LPS-induced production of ROS (Figure 4(E,F)). Taken together, these results suggest that BA can restrain intracellular ROS production, consistent with its effect on activation of the JNK pathway.

### BA protects against LPS-induced damage to the liver and lungs

**Figure 5.**
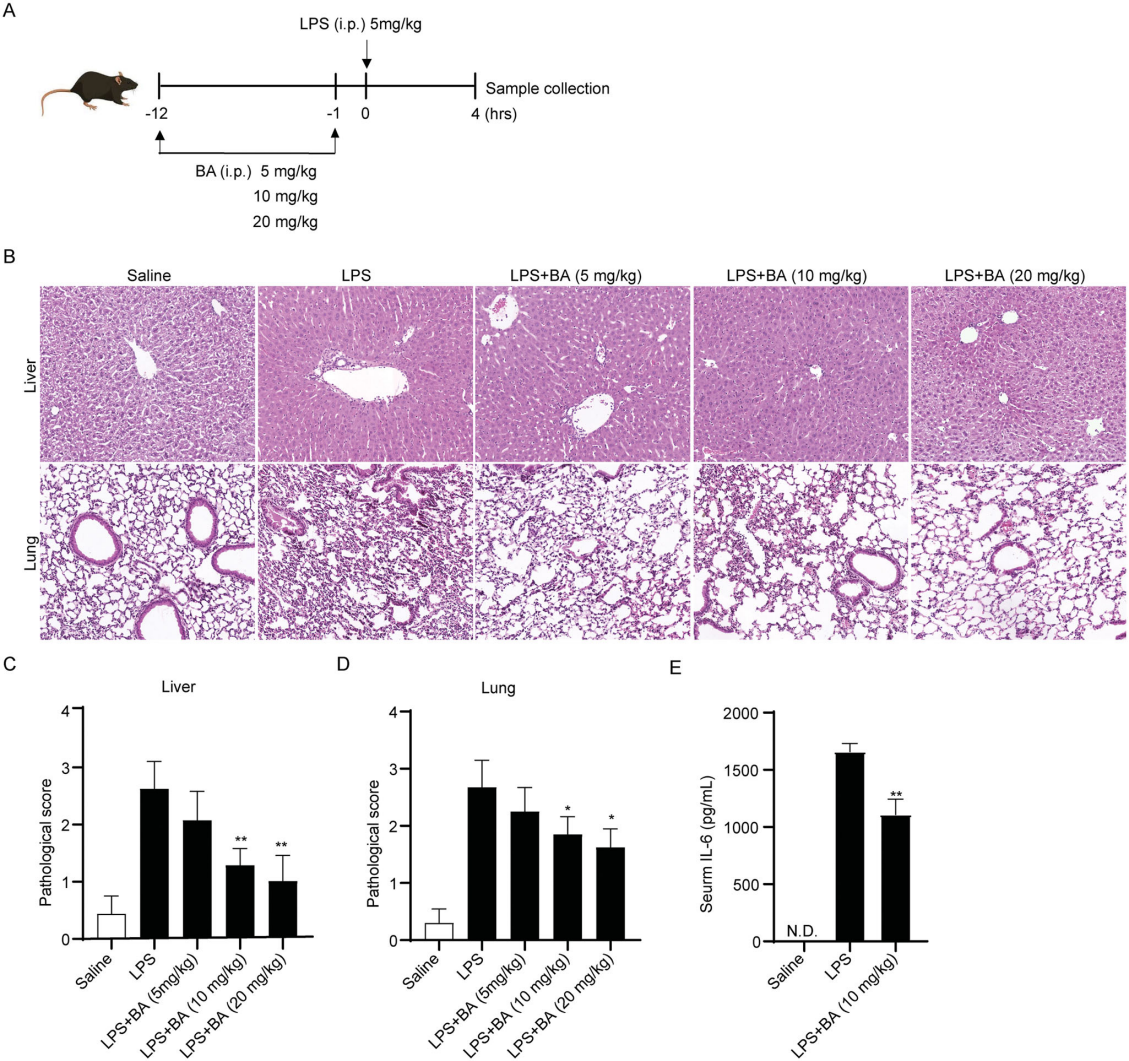
Bullatine A attenuated LPS-induced tissue damage. (A) Schematic graph illustrating the experimental timelines. (B) Representative images of liver and lung H&E staining (scale bars 100 μm). The pathological score of liver (C) and lung (D) (three mice, nine slices each group, data presented as mean ± standard deviation). (E) The serum levels of IL-6 were determined by ELISA (*n* = 5). *p< 0.05 and **p< 0.01 vs. LPS treatment group. N.D.: not detected.

To investigate whether BA pre-treatment could suppress inflammatory responses *in vivo*, we induced the production of systemic inflammatory disturbances in mice via intraperitoneal injection of LPS (Figure 5(A)). The effect of BA on LPS-induced tissue damage was analysed using H&E staining. After LPS injection, the number of infiltrated neutrophils in the liver was significantly increased. However, pre-treatment with BA reduced neutrophil infiltration (Figure 5(B)). Moreover, disruption of alveolar space was observed in the lungs of mice injected with LPS, which was improved by administration of BA (Figure 5(B)). The histological assessment showed that all the above pathological changes induced by LPS were alleviated, and the results were statistically significant when BA at dosages of 10 and 20 mg/kg (Figure 5(C,D)). Moreover, LPS strongly increased the serum level of IL-6 and were also inhibited by BA (Figure 5(E)).

### BA decreased LPS-induced expression of pro-inflammatory cytokines in mice

**Figure 6.**
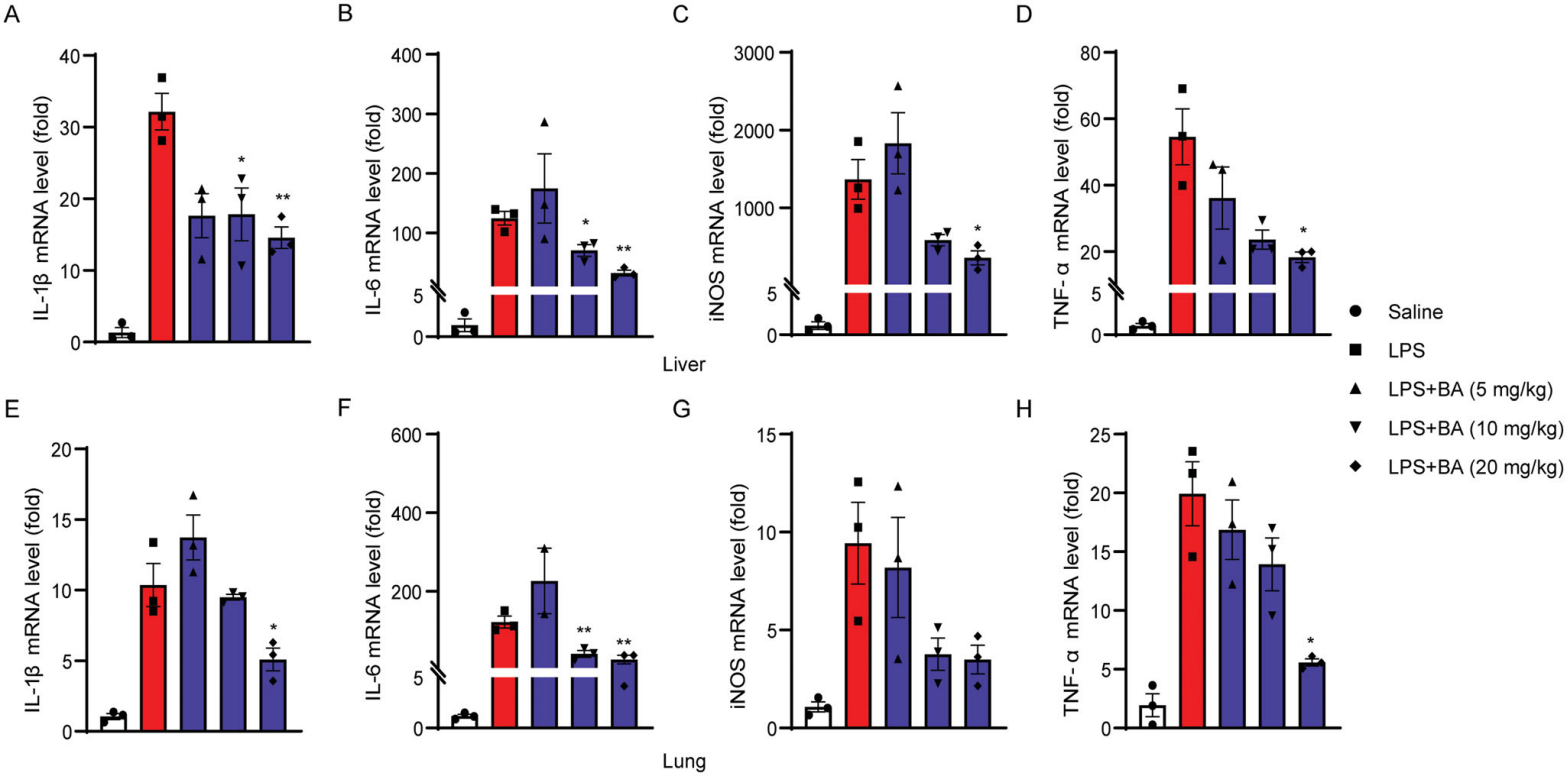
Bullatine A suppressed LPS-induced tissue inflammation. The mRNA level of IL-1β, IL-6, iNOS and TNF-α of liver (A) and lung (B) was measured by quantitative RT-PCR (*n* = 3). *p< 0.05 and **p< 0.01 vs. LPS treatment group.

Next, we investigated whether BA pre-treatment inhibited LPS-induced expression of pro-inflammatory cytokines in the liver and lungs. The results showed that intraperitoneal injection of LPS induced the upregulation of inflammation-related genes (such as IL-1β, IL-6, iNOS and TNF-α) in the liver of model mice, consistent with the induction of acute inflammation. However, pre-treatment with BA (10 and 20 mg/kg) significantly reduced the expression of the above indicators (Figure 6(A–D)). A similar effect was observed in the lungs, although the difference was not statistically significant (Figure 6(E–H)). These results indicate that BA pre-treatment also exerts favourable anti-inflammatory effects *in vivo*.

## Discussion

In recent decades, many studies have examined natural compounds isolated from plants and demonstrated their advantages in treating diseases due to their relatively low toxicity and side effects (Mastinu et al. 2021). In addition, identification of the bioactive compounds in Chinese traditional herbal medicines could help improve our understanding of their pharmacological effects. BA, a major component extracted from *Aconiti brachypodi* Radix, has various pharmacological activities. It has been reported that BA can selectively inhibit expression of the P2X7 receptor and ATP-mediated inflammatory response, suggesting its anti-inflammatory potential (Li J et al. 2013). Herein, we revealed that BA could restrain the LPS-induced inflammatory response both *in vitro* and *in vivo*; the underlying anti-inflammatory mechanism may involve inhibition of the ROS/JNK/NF-κB pathway.

Macrophages and microglia, innate immune cells in the body’s defence system, play an essential role in the inflammatory response and participate in the acute phase of inflammatory diseases by releasing pro-inflammatory cytokines (Wu et al. 2017; Li CL et al. 2019). However, an excessive release of these cytokines can induce serious tissue injury. Therefore, therapies that inhibit the excessive release of pro-inflammatory cytokines are of great significance in inflammatory diseases. In the current study, application of BA reduced the excess expression of inflammatory factors in LPS-activated macrophages and microglia.

The NF-κB pathway is one of the major pathways activated during the inflammatory response, which initiates the transcription of pro-inflammatory factors. Its dysfunction has been associated with many chronic diseases, including asthma, cancer, diabetes, rheumatoid arthritis, inflammation and neurological disorders, and is therefore considered a potential therapeutic target for the treatment of inflammation (Kim et al. 2006; Zhu et al. 2017; Kunnumakkara et al. 2020). Nuclear translocation of p65 is a key signal for NF-κB activation (Liang et al. 2018). In the resting condition, NF-κB is bound to its inhibitor protein IκBα, which restrains it in the cytoplasm (Zhen et al. 2017). Upon stimulation, IκB kinase engages in phosphorylation of NF-κB p65 and ubiquitin-mediated degradation of this product via the proteasome pathway and then translocates p65 into the nucleus, where it triggers the transcription of specific target genes such as IL-1β, IL-6 and TNF-α (Lai et al. 2017; Tang et al. 2017). In this study, BA inhibited IKK phosphorylation and IκB-α degradation, resulting in suppressed nuclear translocation of NF-κB p65 that ultimately impaired activation of the NF-κB pathway.

Several signalling pathways regulate activation of the NF-κB pathway, including the ERK, JNK and p38 pathways (Park JY et al. 2017; Zhang et al. 2019). Here, we revealed that BA significantly inhibited the phosphorylation of JNK, but not that of p38 and ERK1/2. Further studies have suggested that these phenomena may be due to BA decreasing ROS production. ROS have been reported to potentiate several innate immunity pathways to promote the expression of inflammatory genes (Schreck et al. 1991; Cheng et al. 2017; Zhou et al. 2017; Liao et al. 2020). Inhibition of LPS-induced ROS production could decrease the activation of MAPKs, including p38, JNK and ERK, and attenuate activation of the NF-κB pathway (Park J et al. 2015). This is consistent with our findings that LPS exposure leads to increased ROS in macrophages, which is attenuated by BA, suggesting that BA exerts anti-inflammatory activity by restraining oxidative stress.

The LPS is a classical pathogen-associated molecular pattern, which is applied to imitate microbial infection-induced inflammation *in vitro* and *in vivo* (Kong et al. 2020; Wang, Gu, et al. 2021). For *in vivo* study, LPS invades the blood circulation and causes an uncontrolled inflammatory response in the body in a short time, resulting in damage to multiple organs such as liver and lung (Wang, Tan, et al. 2021). In this study, we induced a moderate systemic inflammatory response in mice by intraperitoneal injection of a non-lethal dose of LPS (5 mg/kg). The results showed that BA not only improved the structural damage and inflammatory cell infiltration of liver and lung tissues, but also significantly reduced the levels of pro-inflammatory cytokines in the liver, lung tissues and blood from LPS-treated mice. These findings revealed that BA could effectively alleviate the symptoms of LPS-induced systemic inflammatory response in mice.

## Conclusions

Our study demonstrated that BA effectively inhibits the expression of pro-inflammatory factors by inactivating the ROS/JNK/NF-κB signalling pathway. More importantly, BA preconditioning attenuates the inflammatory response and tissue injury in mice. These findings provide a theoretical basis for the clinical application of BA in the treatment of periphery inflammatory diseases.

## Author contributions

SHL performed the experiments and wrote the manuscript. NC, WO and MCY performed the experiments. YJL and YC conceived and designed the experiments and reviewed the manuscript.
